# A Rapid Method to Measure Serum Retinol Concentrations in Japanese Black Cattle Using Multidimensional Fluorescence

**DOI:** 10.1007/s10895-020-02640-w

**Published:** 2020-10-22

**Authors:** Yoshio Tamura, Hiroki Inoue, Satoshi Takemoto, Kazuo Hirano, Kazutoshi Miyaura

**Affiliations:** Zennoh Central Research Institute for Feed and Livestock, 1708-2 Tsukuriya, Tsukuba, Ibaraki 300-4204 Japan

**Keywords:** Multidimensional fluorescence, Parallel factor analysis, Partial least square regression, Retinol, Serum

## Abstract

Vitamin A levels in fattening Japanese Black cattle affect meat quality; therefore, it is important to monitor serum retinol concentrations. To simplify and accelerate the evaluation of serum retinol concentrations in cattle, we developed a new predictive method using excitation-emission matrix (EEM) fluorescence spectrophotometry. For analytical comparison, the concentration of serum retinol was also measured using the conventional HPLC method. We examined excitation (Ex) and emission (Em) wavelengths of cattle serum, which were 250–450 and 250–600 nm, respectively. Parallel factor analysis separated four components from EEM data, one of which was related to retinol. Next, a partial least square regression model was created using the obtained EEMs as explanatory variables and accrual measurement values as objective variables. The determination coefficient value (*R*^2^), root mean squared error of prediction (RMSEP), and the ratio of performance to deviation (RPD) of the model were determined. A comparison with reference values found that *R*^2^, RMSEP, and RPD of the calibration model were 0.95, 6.4 IU/dl, and 4.2, respectively. This implies that EEM can estimate the serum retinol concentration with high accuracy. Additionally, the fluorescent peaks that contributed to the calibration, which were extracted from the regression coefficient and variable importance in projection plots, were Ex/Em = 320/390 and 330/520 nm. Thus, we assume that this method observes not only free retinol, but also retinol-binding protein. In conclusion, multidimensional fluorescence analysis can accurately and quickly determine serum retinol concentrations in fattening cattle.

## Introduction

Vitamin A is often restricted in the feed of fattening Japanese Black cattle. This improves beef meat quality, increasing marbling, decreasing subcutaneous fat, and increasing the loin eye area [1–3]. However, excessive vitamin A restriction can induce hypovitaminosis A, which results in growth stunting, low immunity, and night blindness [4, 5]. Therefore, vitamin A in fattening Japanese Black cattle needs to be carefully monitored.

Vitamin A deficiency can be detected by measuring the serum retinol concentration. This is commonly done by taking an animal blood sample, pretreating it with an organic solvent, and analyzing it using high performance liquid chromatography (HPLC) [6]. However, HPLC is time intensive and has a high running cost. An alternative method that is simple and quick is desirable. Direct fluorescence has been used to determine serum retinol concentrations in human blood [7, 8]. However, the method is complex, requiring a hundred-fold dilution in 0.1 M NaCl solution. Hence, we propose that simplification of sample preparation will lead to high-throughput monitoring of the serum retinol concentration.

Recently, multidimensional fluorescence has been used to detect metabolites in biological samples to evaluate the quality of various animal products [9–12]. Multidimensional fluorescence is comprehensive, has a high sensitivity, and requires minimal sample preparation steps. It has been used to directly measure biological samples and agricultural products that consist of many compounds. Front-face fluorescence spectroscopy coupled with multivariate analysis has been used to measure *β*-carotene, tryptophan, vitamin A (retinol), and riboflavin concentrations in milk and dairy products [13–16]. Multidimensional fluorescence has also been used with human blood, to quantify riboflavin, aromatic amino acids, and coenzymes (i.e. NADH and FAD) [17–19]. Therefore, this method could potentially be applied to estimate the retinol concentration in cow serum. The aim of this study was to develop a simple high-throughput monitoring tool for serum retinol using multidimensional fluorescence. This will help monitor the health status of fattening Japanese Black cattle.

## Materials and Methods

### Cattle Serum Samples

This study was conducted from June to October, 2019, using fattening Japanese Black cattle bred at Yuzukami farm (Tochigi, Japan), according to “standards relating to the care and keeping of industrial animals” by the Ministry of the Environment, Government of Japan. Blood samples were collected from the jugular veins of 171 castrated males and 37 females (approximately 8–27 months old; Table 1), using VENOJECT II needles (Terumo medical Corp., Tokyo, Japan) and 8-ml serum separator vacuum tubes (Kyokuto Pharmaceutical Industrial Co., Ltd., Tokyo, Japan). The separating tubes were immediately centrifuged (2000×*g*, 4 °C, 30 min) to separate the upper phase. Serum samples were then stored at −80 °C until further analysis.

**Table 1:** Descriptive statistics for the calibration set and the validation set

		Sample sets	
		Calibration set	Validation set
Number of samples		145	63
Age in months	Mean ± SD	19.8 ± 3.9	20.0 ± 3.3
	Range	8.0–27.0	11.0–26.0
Retinol concentration, IU/dl	Mean ± SD	46.7 ± 25.8	47.1 ± 26.8
	Range	11.0–124.0	12.0–124.0

### HPLC Quantitation of Retinol

Retinol quantitation was conducted at Kotobiken medical laboratories, Inc. (Ibaraki, Japan). Retinol was extracted using 400 μl of methanol and 400 μl of the serum sample. The mixture was centrifuged, and the supernatant was then measured by an HPLC/RF-20Axs (Shimadzu Corp., Kyoto, Japan).

### Excitation-Emission Matrix (EEM) Fluorescence Spectrophotometry

To obtain excitation-emission matrices (EEMs) of serum samples, we used a fluorescence spectrophotometer via a micro-plate reader (F-7100; Hitachi high-tech science Corp., Tokyo, Japan). This instrument was equipped with an automatic filtering attachment to remove multi-order light. The conditions used were as follows: photomultiplier voltage, 375 V; response time, 2 ms; scanning speed, 30,000 nm min^−1^; excitation (Ex) wavelength range, 250–450 nm; emission (Em) wavelength range, 250–600 nm; Ex wavelength intervals, 5 nm; and Em wavelength intervals, 1 nm. The plates used were Nunc F96 MicroWell 96-well black polystyrene plates (Thermo Fisher Scientific Inc., MA, United States). Serum samples were pipetted into the micro-well plates (300 μl/well) without pretreatment. Front-face fluorescence spectroscopy was used with three replicates for each sample, performed at 3 min/scan. Data were pre-processed to eliminate unnecessary effects of Raman scattering and multi-order light from the EEM using software FL solution 4.2 (Hitachi high-tech science Corp., Tokyo, Japan). Processed EEMs were outputted as a two-way data matrix for a partial least square (PLS) regression.

### Statistical Analysis

**Fig. 1:**
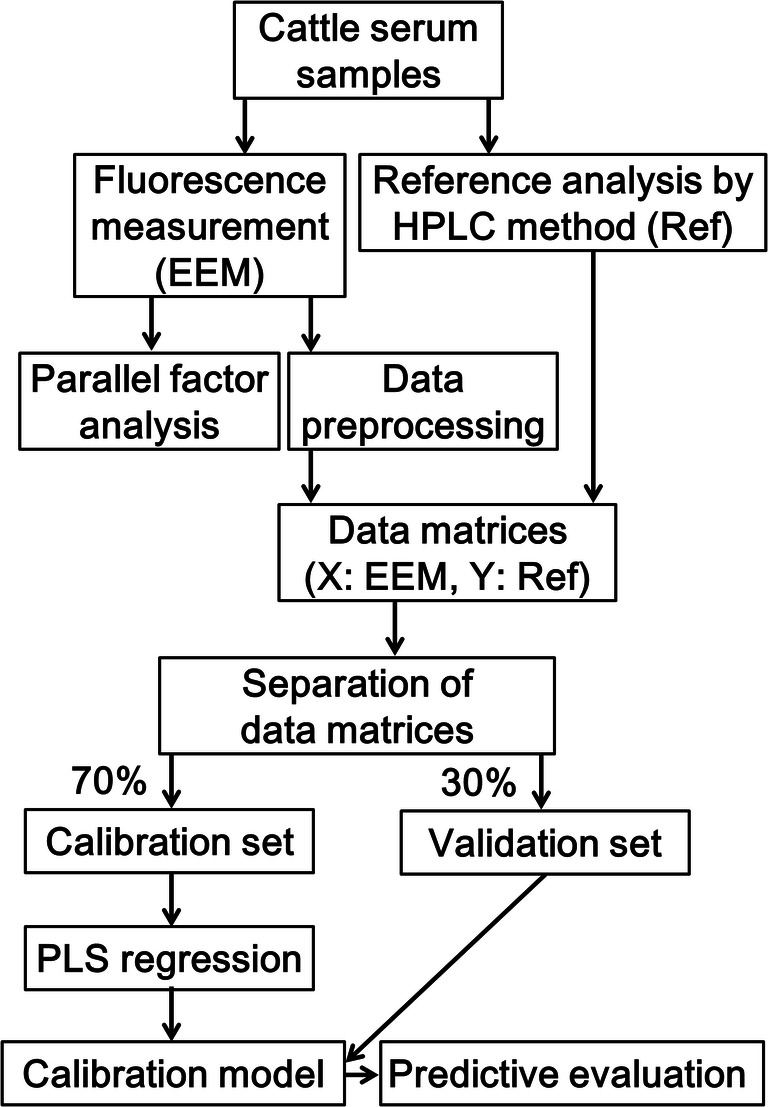
Experimental workflow of serum retinol concentration measurement

To determine significant wavelength ranges of different retinol concentration levels in cattle serum, R packages *EEM* (https://cran.rstudio.com/web/packages/EEM/index.html) and *stardom* (https://cran.r-project.org/web/packages/staRdom/index.html) were used. The *EEM* package was used to make contour plots of non-processed raw data. The *stardom* package was used to separate components of the EEMs with parallel factor analysis (PARAFAC). PARAFAC was conducted for the three-way data array following an excitation × emission × analytical sample of 41 × 351 × 624. Moreover, Pearson’s correlation was calculated with the component score value and reference value to verify whether there is a quantitative relationship between retinol concentrations and their fluorescence intensities. To create a calibration model of serum retinol concentration and determine the prediction ability of this model, all EEMs were randomly divided into calibration (*n* = 145) and validation (*n* = 63) sample sets with three analytical replicates for each sample. The calibration model was built with normalized EEM data (X) and reference values (Y) using PLS regression on SIMCA 15 software (Umetrics, Sweden). The EEM data were normalized using unit variance scaling. The evaluation indices used were coefficient of determination (*R*^2^), root mean square error of evaluation (RMSEE), and root mean square error of prediction (RMSEP). The calibration model had a high *R*^2^ and low RMSEE and RMSEP values, indicating high accuracy. Additionally, the ratio of standard deviation of reference data in the validation set to RMSEP (RPD) was calculated to evaluate the calibration model, where RPD < 1.5 indicates preliminary screening, 2.0–2.5 indicates satisfactory prediction, and > 2.5 indicates good prediction [20–22]. The regression coefficients of the calibration model were indexed to determine which Ex/Em wavelengths contributed positively or negatively to the regression model of the target variable. Moreover, the variable of importance projection (VIP) values greater than 1 were regarded as important to the estimation of the response variable [9, 22]. The workflow of this study is shown in Fig. 1.

## Results and Discussion

### Excitation- and Emission-Matrix Characteristics of Cattle Serum

**Fig. 2:**
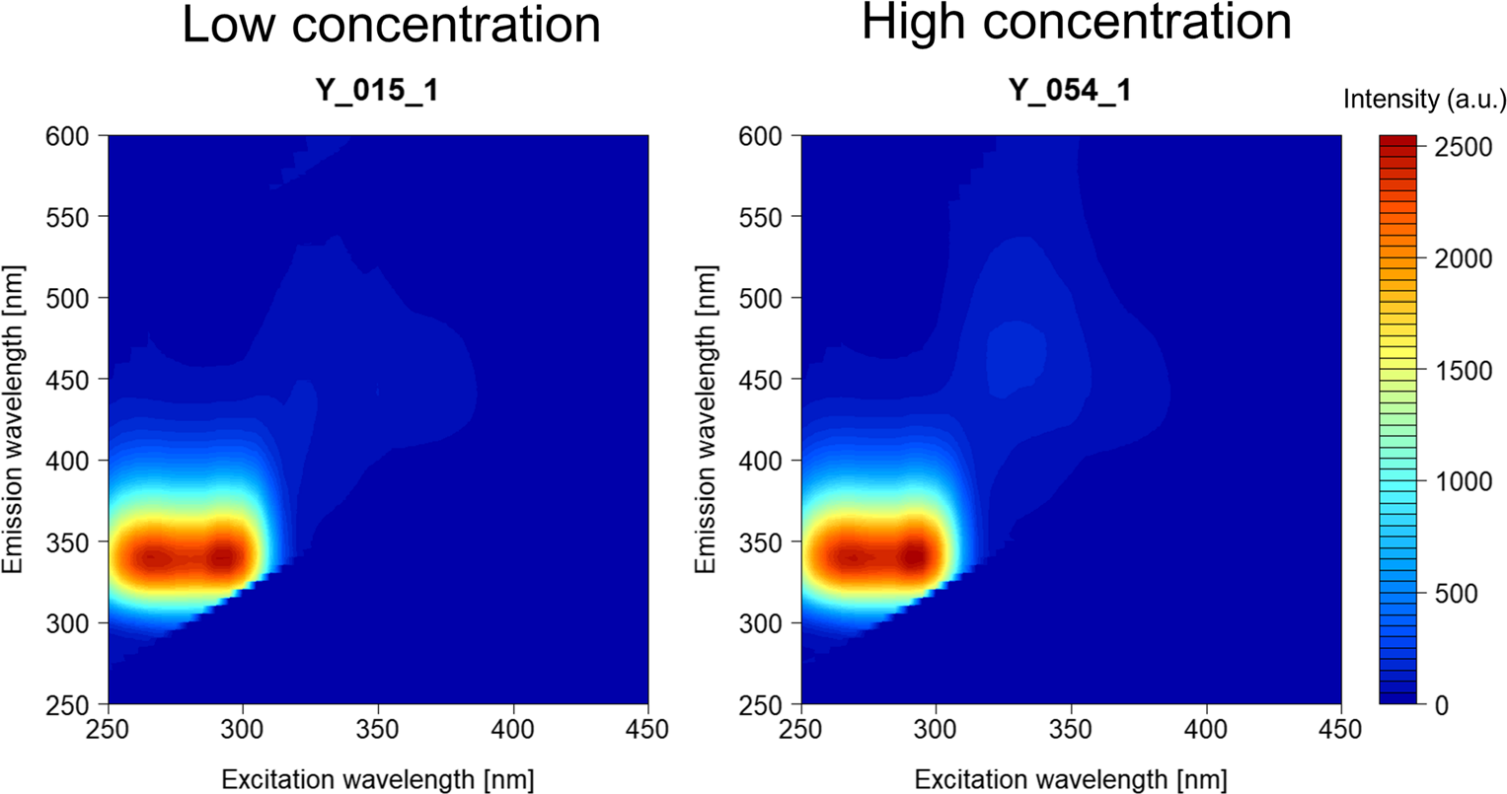
Excitation- and emission-matrices (EEMs) of low and high retinol concentration levels in cattle serum samples. The excitation and emission wavelength intervals were 5 and 1 nm, respectively

**Fig. 3:**
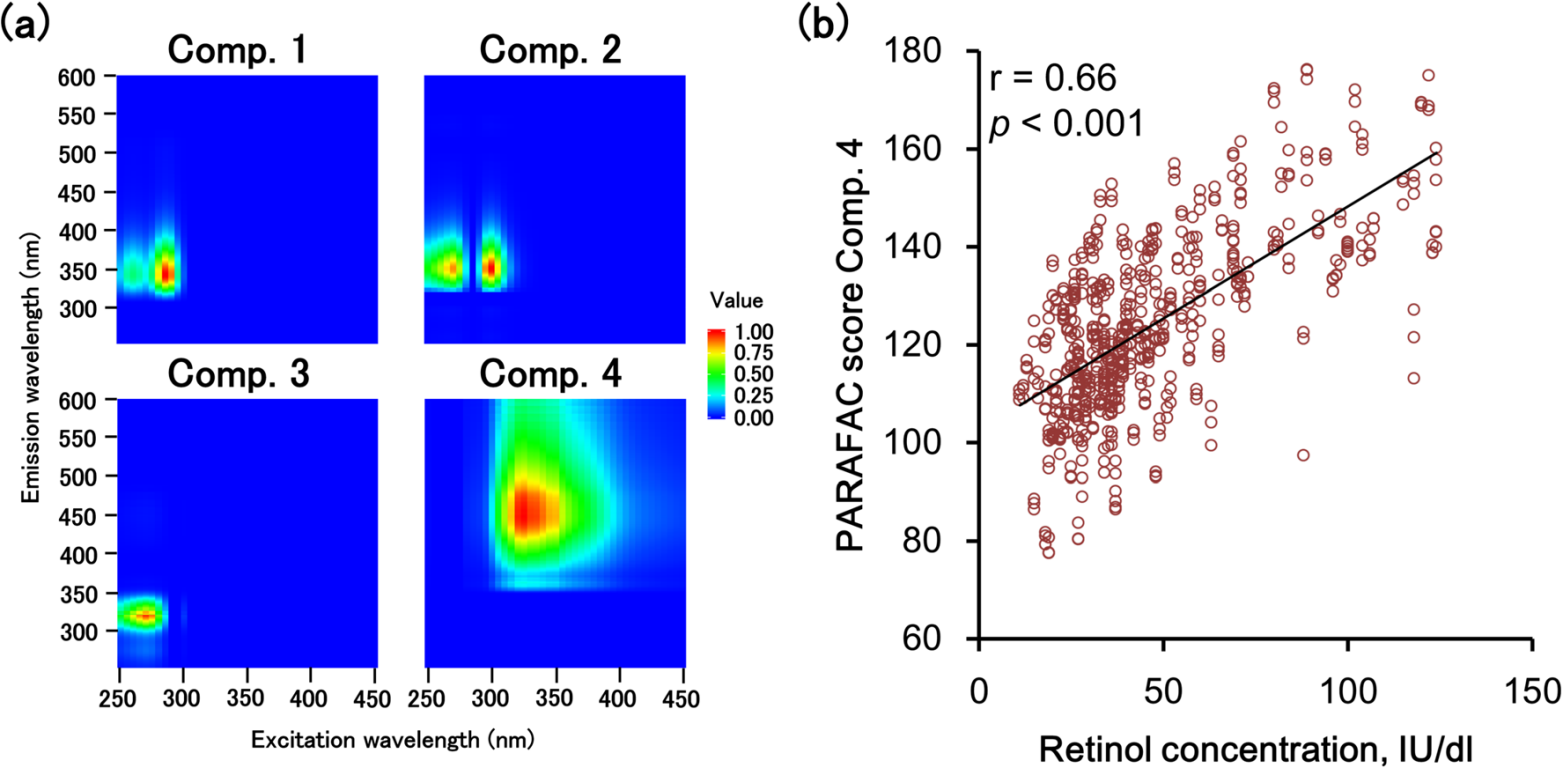
**a** Excitation- and emission-matrix (EEM) spectra of cattle serum decomposed using parallel factor analysis (PARAFAC). **b** Pearson’s correlation between PARAFAC component 4 score values and reference values of serum retinol

The HPLC quantitation results of retinol based on calibration and validation sample sets are shown in Table 1. To investigate EEM characteristics associated with retinol and retinol-related compounds, raw EEMs of cattle serum containing high (124.0 IU/dl) and low (11.0 IU/dl) retinol concentrations were contrasted (Fig. 2). Contour plots of these samples showed common peaks at Ex/Em 290/340 nm and 270/340 nm. Two observed peaks almost corresponded with wavelength ranges of tryptophan residues (Ex/Em, 290/300–400 nm) [23]. However, a peak related to retinol could not be confirmed (Fig. 2). Therefore, we conducted PARAFAC to statistically extract EEM characteristics. Previously, PARAFAC analysis has successfully detected fluorophores such as tryptophan, vitamin B6, and riboflavin from EEMs of brined of salted herring [24]. In the present study, PARAFAC modeling extracted four components with maximum excitation and emission loadings of 285/342 nm, 300/350 nm, 270/320 nm, and 325/447 nm (Fig. 3a). However, an Ex/Em of 280–290/340–370 nm has been found to be related to aromatic amino acids such as tryptophan and tyrosine in dairy and meat products [11, 13]. Therefore, we presumed that the first three components we extracted were related to aromatic amino acids in cattle serum. Conversely, retinol in cow-derived milk and cheese samples have been found to emit at 410, 411 and 435 nm when excited at 320 nm using front-face fluorescence [13, 15]. Vitamin-A (retinol) associated with the quality of buffalo milk and camel milk has been detected with a fluorescence emission at 410 nm and 442 nm (excitation at 320 nm and 322 nm), respectively [25, 26]. Considering the information of retinol fluorescence in the previous studies and our experimental results, the fourth component extracted using PARAFAC (325/447 nm) was assigned to retinol in cattle serum. Moreover, we found a significant correlation (*P* < 0.001) between score values derived from the fourth component and retinol concentrations measured using HPLC (Fig. 3b). This confirms that EEM characteristics can be used to predict serum retinol concentrations in fattening cattle.

### Prediction of Serum Retinol Concentration Using Multidimensional Fluorescence

**Fig. 4:**
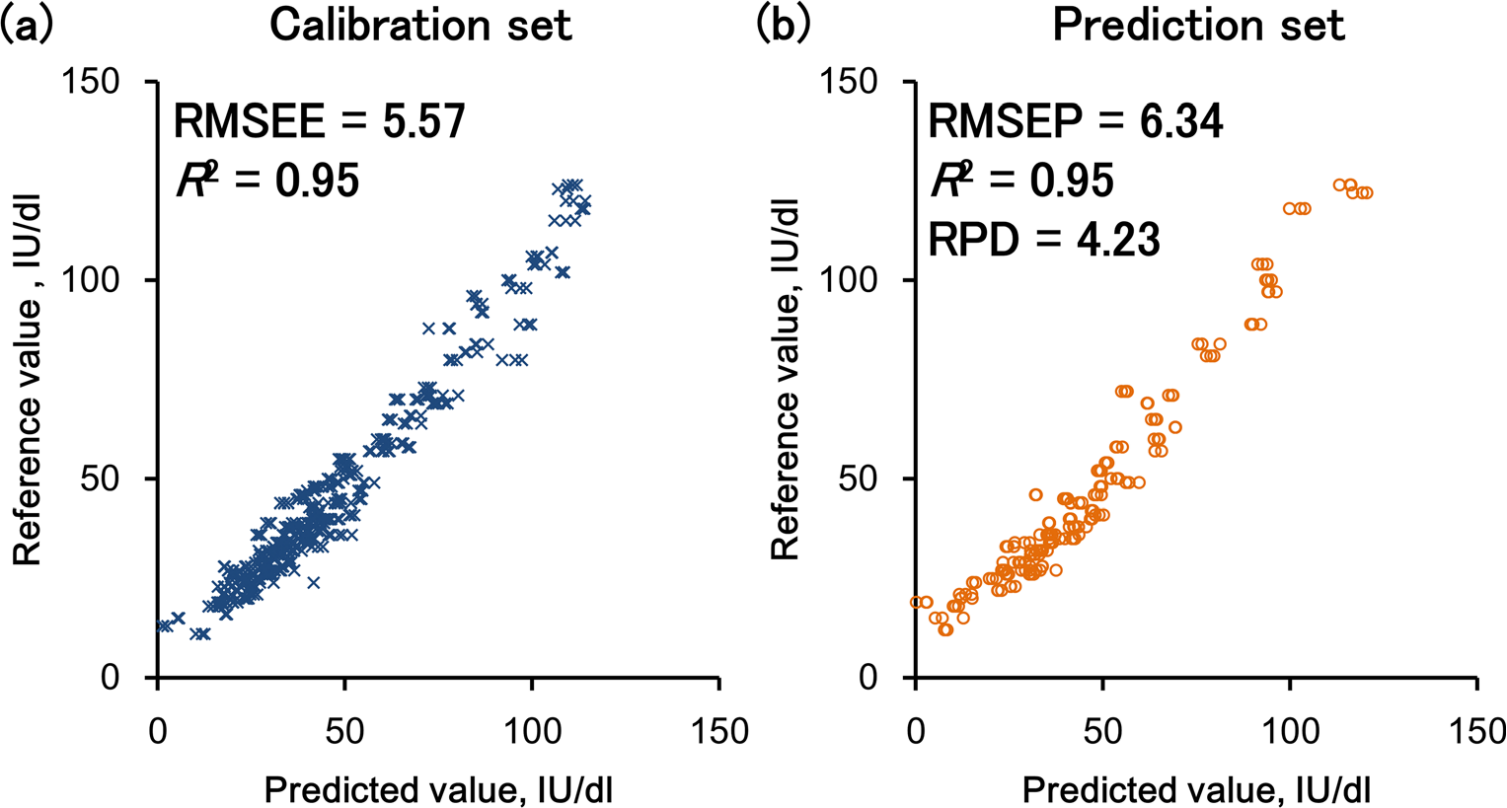
Relationship between reference values and predicted values using a partial least square (PLS) regression model using **a** the calibration data set and **b** the validation data set, where RMSEE is the root mean squared error of evaluation, RMSEP is the root mean squared error of prediction; and RPD is the ratio of performance to deviation

We created a calibration model to predict the serum retinol concentration. Analysis of the model found that it had good accuracy (RMSEE = 5.6, *R*^2^ = 0.95; Fig. 4a) and was robust [intercepts from permutation test were: *R*^2^ = (0.000, 0.005), *Q*^2^ = (0.000, −0.147)] [21, 27]. Next, we evaluated the predictability of the calibration model using the validation sample set. Evaluation indices were as follows: RMSEP = 6.3 IU/dl; *R*^2^ = 0.95; RPD = 4.2 (Fig. 4b). The RPD value exceeded 2.5, indicating that the calibration model can predict serum retinol concentrations with high accuracy.

### Fluorescence Contributions from Serum Retinol in Fattening Cattle

**Fig. 5:**
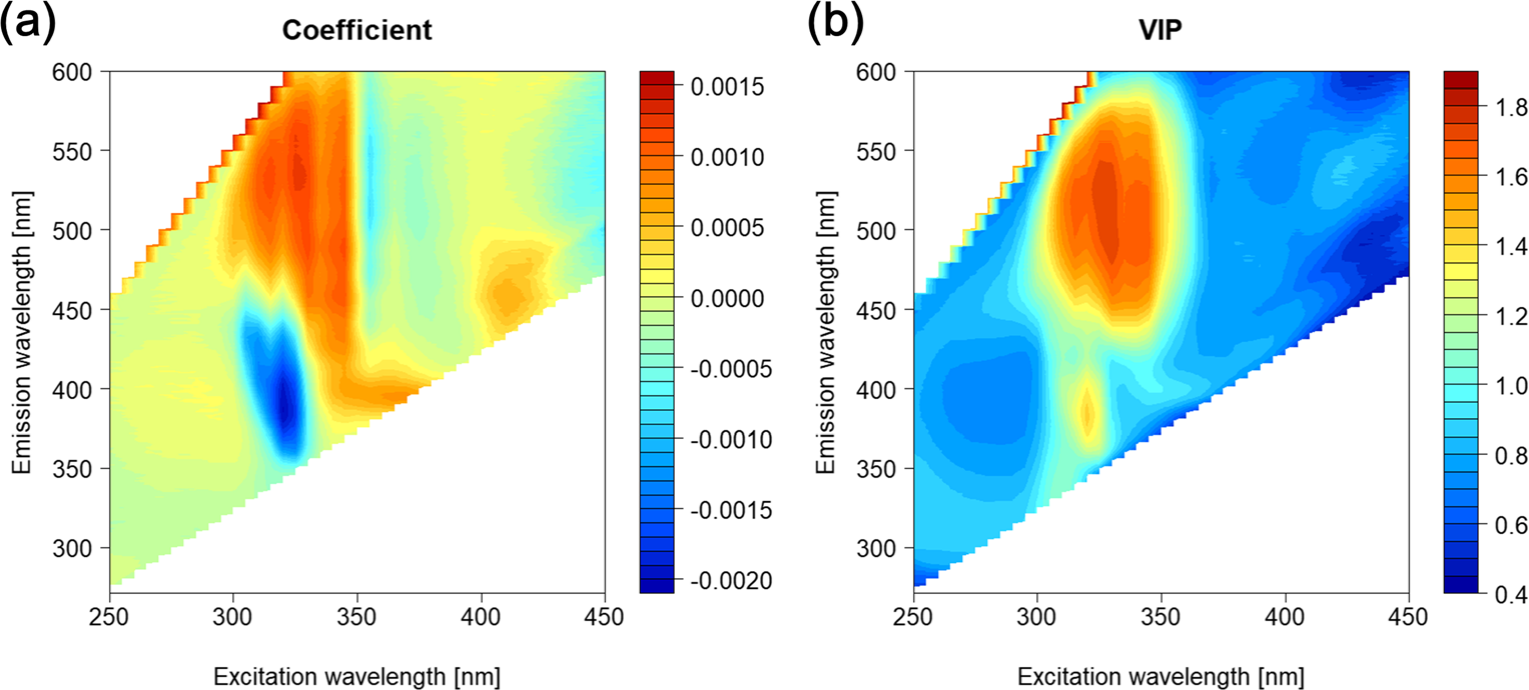
**a** Excitation- and emission-matrix (EEM) contour plots of partial least square (PLS) regression coefficients and **b** variable importance projection (VIP) values

We determined the contribution of Ex and Em wavelengths to serum retinol concentration prediction. Contour plots of the PLS regression coefficients and the VIP values are shown in Fig. 5. Importance variables of the calibration model are usually regarded as VIP >1. The main peaks of the VIP contour plot were at 320/390 nm and 330/530 nm (Fig. 5b), with negative and positive regression coefficients, respectively (Fig. 5a). The peak of 320/390 nm roughly corresponded with the Ex/Em wavelength of retinol [15]. A direct fluorescent measurement of retinol concentrations in human serum diluted with 0.1 M NaCl used a 335 nm excitation and 460 nm emission peak [7, 8]. Furthermore, Watanabe et al. (2008) [28] suggested the measurement of serum retinol using a fluorescence microplate reader with an Ex/Em of 335/510 nm. However, their method targeted retinol-binding protein, which has a higher fluorescence intensity than free retinol. Therefore, in our study, both free and bound retinol might contribute to Ex/Em peaks.

For the first time, we have succeeded in applying multidimensional fluorescence to the estimation of retinol content in cattle serum. Our new method is able to make monitoring vitamin A in fattening Japanese Black cattle easier and cheaper, without cumbersome operations and reagents. However, it might be difficult to apply our regression model to samples from cattle under different conditions that can affect the composition of blood metabolites. For example, heat-stress can affect plasma metabolite profiles (e.g. concentrations of carbohydrates, lipids, and amino acids) in cattle [27]. Additionally, the composition of blood metabolites in cattle finished on grass differs from those finished on grain [29]. Hence, to ensure predictive accuracy, the multidimensional fluorescence technique should be used for cattle serum under regulated conditions such as rearing environments and feeding management.

## Conclusion

We developed a rapid method to directly measure serum retinol concentrations in cattle using multidimensional fluorescence without the need for sample preparation. This provides a practical and highly accurate way to monitor vitamin A intake in fattening Japanese Black cattle to ensure high-quality meat. As previously reported, the EEMs of food and biological samples have been used to measure various compounds such as amino acids, pigments, and vitamins. Therefore, we are confident that the fluorescence method can be used in livestock health checks to measure multiple metabolites in serum.

### Acknowlegements

We thank Mr. Yoichi Kimijima (JA Higashinihon Kumiai Shiryo Co., Japan) for assistance with collecting cattle serum samples and Dr. Yonathan Asikin (University of the Ryukyus) for helpful comments on the manuscript.

## Data Availability

No applicable.
